# Preclinical evaluation of dose-dependent effects of photobiomodulation therapy on persistent inflammation in the temporomandibular joint

**DOI:** 10.22514/jofph.2025.037

**Published:** 2025-06-12

**Authors:** Vinicius Almeida Carvalho, Amanda Almeida Martins, Amanda de Carvalho Desiderá, Glauce Crivelaro Nascimento, Laís Valencise Magri, Christie Ramos Andrade Leite-Panissi

**Affiliations:** ^1^Department of Oral and Basic Biology, Ribeirão Preto Dentistry School, University of São Paulo, 14040-904 Ribeirão Preto, SP, Brazil; ^2^Departament of Restorative Dentistry of Dentistry School of Ribeirão Preto, University of São Paulo, 14040-904 Ribeirão Preto, SP, Brazil; ^3^Department of Psychology, School of Philosophy, Science and Literature of Ribeirão Preto of the University of São Paulo, 14040-901 Ribeirão Preto, SP, Brazil

**Keywords:** Low-level laser therapy, Inflammation, Temporomandibular disorders, Pain

## Abstract

**Background**: Low-level laser therapy (LLLT) is a non-invasive, innovative approach for alleviating pain and improving function in temporomandibular joint (TMJ) disorders. This study aimed to compare the effect of three different doses of photobiomodulation (PBM) (2.5, 5 or 10 J/cm^2^) on the reduction of nociceptive events of rats’ TMJ with persistent inflammation and inflammatory profile modulation. **Methods**: Male Wistar rats (n = 240) were submitted to a model of temporomandibular inflammation induced by induced by Complete Freund’s Adjuvant (CFA) and treated with PBM on days 1, 3, 5, 7 and 10 post-CFA or saline (SAL) injection. Assessments included orofacial mechanical sensitivity through von Frey test, quantification of Evans blue plasma extravasation, myeloperoxidase activity analysis (MPO) and neutrophil/leukocyte infiltration in synovial fluid and TMJ tissues. **Results**: Regarding the analgesic effect of LLLT, an inverted U-shaped curve was observed, as despite the effectiveness of all three tested doses, the 5 J/cm^2^ dose completely prevented the reduction in the mechanical allodynia threshold induced by temporomandibular inflammation. Regarding the evaluation of inflammatory parameters, all three doses studied effectively reduced plasma extravasation, neutrophils and MPO, with more pronounced effects observed at the 5 and 10 J/cm^2^ doses. Interestingly, the lowest dose evaluated, 2.5 J/cm^2^, partially prevented mechanical allodynia, plasma extravasation, MPO activity and neutrophil count, both in the acute application and with repeated treatment for up to 10 days. Total leukocyte levels, however, were reduced with LLLT at the 5 and 10 J/cm^2^ doses. **Conclusions**: This study highlights the dose-dependent efficacy of LLLT, with an intermediate dose (5 J/cm^2^) producing the most pronounced analgesic effects and the 5 and 10 J/cm^2^ doses demonstrating significant therapeutic benefits in reducing acute and persistent TMJ inflammation. Importantly, even the lowest dose effectively modulated key components of the innate immune response, indicating its potential to reduce early inflammatory processes.

## 1. Introduction

Temporomandibular disorders (TMD) cover some clinical conditions affecting the temporomandibular joint (TMJ), masticatory muscles, and related structures [1]. TMD affects approximately 5–12% of the general population, with a higher prevalence among women aged 20 to 40 years [2]. TMD remains a complex group of conditions influenced by genetic, epigenetic, and environmental factors [3]. Current research highlights an intricate interplay of biological mechanisms, including dysregulated nociceptive pathways, immune-neuroimmune interactions and joint mechanics [1].

Pain is one of the most common and limiting clinical manifestations of such disorders [4]. Around 10% of adults experience orofacial pain related to TMD, which significantly impacts their quality of life by affecting physical functionality and contributing to psychosocial distress [4]. Moreover, the multifactorial nature of TMD underscores its heterogeneity. According to the Diagnostic Criteria for TMD (DC/TMD) Axis I, these disorders are categorized into three main groups based on their clinical presentation. Group I encompasses muscle disorders, including myofascial pain, with or without limitations in mouth opening. Group II involves joint-related conditions, such as displacement, with or without reduction, and limitations in mouth opening. Finally, Group III includes arthralgia, arthritis, and arthrosis, which are characterized by inflammation or degeneration of the TMJ [5]. About 25–55% of TMD patients exhibit degenerative changes in the TMJ, often linked to poor clinical outcomes [6]. Research focuses on the relationship between joint degeneration and pain, aiming to improve management strategies and develop regenerative therapies.

Alternative therapies, such as Photobiomodulation Therapy (PBM), have shown promising effects in attenuating the symptoms of TMDs, and numerous examinations have affirmed the pain-relieving and anti-inflammatory effects of PBM in both experimental [7, 8] and clinical trials [9, 10, 11]. Arthrogenous TMD, which encompasses conditions such as arthralgia, arthritis and arthrosis, is often managed using conservative, non-invasive therapeutic approaches aimed at alleviating pain and restoring function. Among these, PBM, commonly referred to as laser therapy, has gained significant attention due to its anti-inflammatory, analgesic and regenerative properties. Its capacity to promote collagen synthesis and cartilage repair highlights its potential role in addressing the degenerative aspects of TMJ arthrosis. Compared to pharmacological interventions, laser therapy offers several advantages, including minimal side effects, non-invasiveness and the ability to target localized tissues effectively [12].

Current evidence suggests that PBM may reduce inflammation by influencing key cellular and molecular targets [8]. Photobiomodulation reduces inflammation by lowering pro-inflammatory cytokines such as tumor necrosis factor-alpha (TNF-α) and interleukin-6 (IL-6), while increasing the levels of the anti-inflammatory cytokine interleukin-10 (IL-10). It inhibits cyclooxygenase-2 (COX-2) activity, decreases prostaglandin E2 (PGE2) levels, and reverses pain sensitivity by reducing the expression of substance P, transient receptor potential vanilloid 1 (TRPV-1), and calcitonin gene-related peptide (CGRP) in TMJ lesions. Photobiomodulation also reduces oxidative stress by enhancing mitochondrial function and adenosine triphosphate (ATP) production, lowering reactive oxygen species (ROS), and improving cellular resilience [7, 9].

Clinical studies in patients with TMJ disorders have highlighted PBM’s efficacy in reducing pain intensity, improving mandibular range of motion, and enhancing overall functionality [13]. In fact, laser therapy effectively reduces pain in patients with both myogenic and arthrogenic TMD [12]. Despite this, there has yet to be a scientific consensus about the dosages and protocols of application, and clinical outcomes still need to be better and more predictable [14, 15, 16, 17, 18, 19].

Despite these promising findings, the variability in PBM protocols, including differences in wavelength, dosage and treatment frequency, complicates the direct comparison of results across studies. Standardization of these parameters, alongside further investigations into the cellular and molecular mechanisms of PBM, are essential to optimize its clinical application in TMJ disorders and fully elucidate its anti-inflammatory potential. This study aimed to compare the effect of three different doses of low-level laser therapy (2.5, 5 or 10 J/cm^2^) on the reduction of nociceptive events of rats’ TMJ with persistent inflammation.

## 2. Materials and methods

### 2.1 Animals

Wistar male rats (200–250 g) were used to perform experiments. They were housed in a room with a 12 h light/dark cycle with food and water ad libitum and controlled temperature (24 °C). The experimental procedures were approved by the Animal Care and Use Committee of the University of São Paulo, Ribeirão Preto (protocol # 11.1.888.53.5 # 2014.1.509.58.8). All efforts were made to ensure minimal animal suffering and to reduce the number of animals used in this study. Rats were randomly assigned to treatment groups using a computer-generated randomization process. The experiments were conducted double-blind, ensuring data collection and analysis were performed without bias. Statistical power was set at 80% to ensure the robustness of the findings.

### 2.2 Complete Freund’s adjuvant (CFA) administration

In anesthetized rats (ketamine and xylazine—75 and 10 mg/kg, respectively, administered intramuscularly), CFA intraarticular administration was performed. Using a micro syringe (Hamilton model 705RN; Hamilton, Reno, NV, USA) coupled to a 30-gauge gingival (BD, Franklin Lakes, NJ, USA), 50 μg of CFA suspended in a 50 μL paraffin oil (18512, Sigma, St. Louis, STL, USA) or 0.9% saline (SAL) was applied bilaterally on TMJ (into the supra-discal space). The confirmation of the local was verified by moving the mandible, and the puncture of the needle into the joint space was confirmed by the loss of resistance [20]. 1, 3, 5, 7 or 10 days after the administration of CFA or SAL, TMJ and synovial fluid were collected for plasma extravasation analysis, neutrophils counting, leukocyte differential counting and myeloperoxidase activity.

### 2.3 Low-level laser therapy (LLLT)

A low-level intensity infrared laser (gallium aluminum arsenide semiconductor diode laser device, Laser Twin Set MM Optics, São Carlos, SP, Brazil) with a gallium-aluminum-arsenide semiconductor (GaAlAs) was used. After the administration of CFA or SAL, the LLLT session took place (day 1). LLLT was repeated on days 3, 5, 7 and 10. The energy dose used were 2.5 J/cm^2^, 5 J/cm^2^ or 10 J/cm^2^ (respectively, 5 mW/10 s, 8 mW/10 s, 10 mW/10 s, λ = 780 nm, 0.04 cm^2^), at only one point on the TMJ.

### 2.4 Orofacial mechanical sensitivity

The orofacial sensitivity was evaluated by the head withdrawal reflex during the application of the mechanical stimuli before and 1, 3, 5, 7 and 10 days after CFA or SAL was administered into the TMJ by the von Frey test. To measure the mechanical sensitivity, rats were placed in the testing chamber and a progressive force from the filament of an electronic von Frey anesthesiometer (EFF 301 Analgesímetro Digital Von Frey, Insight Instruments, Ribeirao Preto, SP, Brazil) was applied to the TMJ region. The withdrawal threshold head was calculated as the mean ± standard error of the mean (SEM) based on three values obtained in each session [21].

### 2.5 Euthanasia

The rats were sacrificed by cervical dislocation. The facial skin was excised, and the temporal muscle overlying the TMJ was carefully dissected. A 30-gauge needle was inserted through the posterior membrane, and the synovial cavity was washed by injecting and immediately aspirating 50 μL of phosphate-buffered saline (PBS) plus ethylenediaminetetraacetic acid (EDTA) solution (10 mmol/L). The washing procedure was repeated, and the collected fluids were kept at 70 °C until MPO activity and cell counting were performed. The dissected temporomandibular tissue was used for the quantification of Evans blue extravasation.

### 2.6 Quantification of Evans blue extravasation

Plasmatic extravasation on TMJ was measured by intravenous injection of Evans blue dye (25 mg/kg) 30 min before euthanasia on the last experiment day. After transcardiac perfusion with PBS solution, the periarticular tissue was dissected, weighed and kept in 2 mL of formaldehyde overnight. The supernatant (100 μL) was extracted, and it was read by an absorbance at 630 nm in a spectrophotometer. Dye concentrations were compared with a standard curve of known amounts of Evans blue dye. The quantity of Evans blue dye (μg) per mL of rat tissue exudate was calculated [22].

### 2.7 Myeloperoxidases activity analysis (MPO)

This study conducted MPO assays on synovial fluid from temporomandibular joints in distinct rat groups. Briefly, 20 μL of synovial fluid was homogenized in 1 mL of hexadecyltrimethylammonium bromide (HTAB) and this homogenate was centrifuged at 4500 rpm for 12 min at 4 °C. In the resuspended pellet, MPO activity was assayed by measuring the change in absorbance at 450 nm using o-dianisidine dihydrochloride and 1% hydrogen peroxide. A unit of MPO activity was defined as the conversion of 1 μmol of hydrogen peroxide to water in 1 min at 22 °C. The results are reported as MPO units/joint (μL).

### 2.8 Inflammatory cells counting

The present protocol was based on a previous study [23]. 20 μL of synovial fluid from temporomandibular joints collected from each animal were diluted with 380 μL of Turk solution (1:20 dilution). The total number of white cells was determined using a hemacytometer (Neubauer chamber). For neutrophil count, slides were prepared using an aliquot of the washed joint fluid (50 μL). The slides were stained with eosin and hematoxylin and the counting was performed in an optical microscope with a 40× objective. The results are expressed as the number of cells ×10^6^/mL.

### 2.9 Experimental design

Starting on day 0, all animals underwent a 7-day acclimatization period. Baseline nociceptive assessments using the Von Frey test were conducted on the seventh day (designated as day 0). Following this initial measurement, animals received intra-articular injections of either saline or CFA in both temporomandibular joints (left and right).

The animals were then divided into eight experimental groups:

1. Sham: Saline injection with no photobiomodulation therapy (LLLT).

2. SAL 2.5 J/cm^2^, SAL 5 J/cm^2^, SAL 10 J/cm^2^: Saline injection followed by LLLT at doses of 2.5 J/cm^2^, 5 J/cm^2^ or 10 J/cm^2^.

3. CFA: CFA injection with no LLLT.

4. CFA 2.5 J/cm^2^, CFA 5 J/cm^2^, CFA 10 J/cm^2^: CFA injection followed by LLLT at doses of 2.5 J/cm^2^, 5 J/cm^2^ or 10 J/cm^2^.

Independent groups were assessed for the five post-CFA and treatment time points: 1, 3, 5, 7 and 10 days. The Von Frey test was performed 60 minutes after each LLLT application. After each time point (days 1, 3, 5, 7 and 10), animals were euthanized immediately after the Von Frey test. Synovial fluid and temporomandibular joint tissues were then collected for molecular analysis.

### 2.10 Statistical analysis

Results are shown as the mean ± the SEM. A two-way analysis of variance (ANOVA) was used, with time and treatment as factors for the statistical analysis. For the von Frey test, a repeated-measures two-way ANOVA was used. These tests were followed by the Newman-Keuls test. A significance of *p* < 0.05 was considered statistically different.

## 3. Results

A total of eight experimental groups (n = 6) were used in this study for each analyzed time point (five time points), resulting in a total of 240 rats.

### 3.1 Orofacial mechanical sensitivity

The results demonstrate that LLLT with 5 and 10 J/cm^2^ in rats with inflammation induced by CFA abolished orofacial mechanical allodynia (Fig. 1). In the Saline and Saline + LLLT groups there was no significant decrease in the mechanical threshold during all the experimental times. A two-way ANOVA Repeated Measures (RM) revealed a significant decrease in mechanical threshold by time (*F*_(4, 139)_ = 14.938, *p* < 0.001) and treatment (*F*_(4, 139)_ = 24.78, *p* < 0.05) and an interaction between treatment and time (*F*_(12, 139)_ = 3.723, *p* < 0.001). The Newman-Keuls test (*p* < 0.05) revealed a difference among the basal threshold measure and all periods analyzed in the CFA and CFA + 2.5 LLLT groups. However, in LLLT 5 and 10 J/cm^2^ there were no significant reductions in head withdrawal thresholds in rats with temporomandibular inflammation over the periods compared to the respective basal thresholds (Fig. 1). Also, the head withdrawal thresholds of the CFA + 5 J/cm^2^ and CFA + 10 J/cm^2^ groups did not differ from the Saline and Saline + LLLT groups (Fig. 1).

**Fig. 1.** Time course of mechanical sensitivity. The mechanical nociceptive threshold was measured before and 1, 3, 5, 7 and 10 days after saline (SAL) (A) or CFA (B) injection into the TMJ and low-level laser therapy at different doses (2.5, 5 or 10 J/cm^2^). The data are expressed as means ± SEM (n = 6 per group). #*p* < 0.05 for the Newman-Keuls method when comparing the CFA and CFA + 2.5 LLLT groups with CFA + 5.0 LLLT and CFA + 10 LLLT experimental groups. SEM: standard error means; CFA: Complete Freund’s Adjuvant.

### 3.2 Plasma extravasation in TMJ

CFA administration to the TMJ region increased plasma extravasation in the temporomandibular tissues which was reduced by LLLT (Fig. 2). A two-way ANOVA revealed the effects of treatment (*F*_(4, 143)_ = 7.35, *p* < 0.001) and time (*F*_(3, 143)_ = 36.87, *p* < 0.001) and interaction between treatment and time (*F*_(12, 143)_ = 3.44, *p* < 0.01). The 2.5 J/cm^2^ dose reduced the plasma extravasation 7 and 10 days after TMJ inflammation (Fig. 2, Newman-Keuls, *p* < 0.05). Regarding the 5 J/cm^2^ dose, CFA groups had decreased plasma extravasation in 3-, 5-, 7- and 10-days periods compared to the first day of inflammation (Newman-Keuls, *p* < 0.001, Fig. 2). Considering 10 J/cm^2^ dose, there were no statistical differences between the CFA groups compared to the Saline groups (Fig. 2).

**Fig. 2.** Evaluation of plasma extravasation based on Evans Blue dye in the TMJ tissues of the rats after different periods following the administration of CFA or Saline (Sham) and LLLT at doses of 2.5 J/cm^2^, 5 J/cm^2^ and 10 J/cm^2^. The data are expressed as means ± SEM (n = 6 per group). *p < 0.05 using the Newman-Keuls method when comparing the CFA and Saline groups. #*p* < 0.05 using the Newman-Keuls method when comparing the CFA group at 7 and 10 days with PT at 2.5 J/cm^2^ with the CFA groups in the other periods using the same laser dose. +*p* < 0.05 using the Newman-Keuls method when comparing the CFA group with 1-day laser treatment (5 J/cm^2^) to the CFA groups in the other periods using the same laser dose. SEM: standard error means; CFA: Complete Freund’s Adjuvant.

### 3.3 MPO activity

The CFA increased MPO levels in the TMJs synovial fluid (Fig. 3) but LLLT reduced these levels in the inflamed tissue (Fig. 3). A two-way ANOVA revealed the effects of treatment (*F*_(3, 122)_ = 742.66, *p* < 0.001) and time (*F*_(3, 122)_ = 145.48, *p* < 0.001), as well as an interaction between treatment and time (*F*_(3, 122)_ = 145.48, *p* < 0.001). Compared to Saline groups, the CFA groups increased in MPO levels over time and at 1, 3, 5 and 7 days after 2.5 J/cm^2^ dose (Newman-Keuls, *p* < 0.05). At 10 days of 2.5 J/cm^2^ dose, the CFA group and Saline group did not differ statistically (Fig. 3). LLLT of 5 or 10 J/cm^2^ doses had a positive effect in the TMJ during all of the periods analyzed after CFA-induced inflammation since Newman-Keuls test did not reveal a difference in MPO activity among the inflamed and not inflamed groups (Fig. 3).

**Fig. 3.** Evaluation of myeloperoxidase (MPO) activity in the synovial fluid of the rats after different periods following the administration of CFA or Saline (Sham) and LLLT at doses of 2.5 J/cm^2^; 5 J/cm^2^ and 10 J/cm^2^. Data are expressed as means ± SEM (n = 6 per group). *p < 0.05 Newman-Keuls when comparing the CFA and Saline groups. SEM: standard error means; CFA: Complete Freund’s Adjuvant.

### 3.4 Inflammatory cell influx

Our results revealed an increase of neutrophils in TMJ fluid, but LLLT reduced these levels in the inflamed tissue (Fig. 4). A two-way ANOVA showed significant differences in treatment (*F*_(1, 135)_ = 16.86, *p* < 0.001) and an interaction between time and treatment (*F*_(1, 115)_ = 14.4, *p* < 0.001). At 10 days, there was no difference between the Saline and CFA groups (Fig. 4); also, the CFA + 2.5 J/cm^2^ group presented a decrease in neutrophils influx at this period, compared to the CFA group at all other periods. LLLT at 5 J/cm^2^ dose promoted neutrophil decrease in the CFA groups at all periods, compared to the CFA group at 1 day of LLLT. Also, significant differences existed between the Saline and CFA groups at 1, 3 and 5 days, which were not observed at 7 and 10 days (Fig. 4, Newman-Keuls, *p* < 0.05). LLLT at 10 J/cm^2^ dose, there were no differences in the Saline and CFA groups (Fig. 4).

**Fig. 4.** Evaluation of neutrophil influx based on cell counting in TMJ tissues of the rats after different periods following the administration of CFA or Saline (Sham) and LLLT at doses of 2.5 J/cm^2^, 5 J/cm^2^ and 10 J/cm^2^. The data are expressed as means ± SEM (n = 6 per group). *p < 0.05 using the Newman-Keuls method when comparing the CFA and Saline groups. #*p* < 0.05 using the Newman-Keuls method when comparing the CFA group with 10 days of LLLT at 2.5 and 5 J/cm^2^ doses to the CFA groups in the other periods using the same laser doses. +*p* < 0.05 using the Newman-Keuls method when comparing the CFA group with 1-day laser treatment (2.5 and 5 J/cm^2^) to the CFA groups in the other periods using these same laser doses. SEM: standard error means; CFA: Complete Freund’s Adjuvant.

Regarding leukocyte influx, two-way ANOVA showed significant differences between the treatments (*F*_(1, 47)_ = 433.62, *p* < 0.001) and time (*F*_(3, 47)_ = 35.31, *p* < 0.001), and an interaction between treatment and time (*F*_(3, 47)_ = 2.89, *p* < 0.05). The CFA groups differed from the Saline groups and CFA + 2.5 J/cm^2^ in all periods (Fig. 5, Newman-Keuls, *p* < 0.01). When a LLLT at 5 J/cm^2^ was used the same pattern was verified at 1, 3, 5 and 7 days. However, at 10 days, the Saline and CFA + 5 J/cm^2^ groups did not differ and the CFA group presented a significant decrease in leukocyte influx compared to other periods (Fig. 5, Newman-Keuls, *p* < 0.05). Finally, for 10 J/cm^2^ dose, the Newman-Keuls test revealed differences (*p* < 0.05) in leukocyte influx among the CFA + 10 LLLT groups at 3, 5, 7 and 10 days compared with 1 day (Fig. 5).

**Fig. 5.** Evaluation of leukocyte influx based on cell counting in TMJ tissues of the rats after different periods following the administration of CFA or Saline (Sham) and LLLT at doses of 2.5 J/cm^2^, 5 J/cm^2^ and 10 J/cm^2^. The data are expressed as means ± SEM (n = 6 per group). *p < 0.05 using the Newman-Keuls method when comparing the CFA and Saline groups. #*p* < 0.05 using the Newman-Keuls method when comparing the CFA group with 10 days of laser treatment (5 J/cm^2^) to the CFA groups in the other periods using this same laser dose. SEM: standard error means; CFA: Complete Freund’s Adjuvant.

## 4. Discussion

In this study LLLT at 5 and 10 J/cm^2^ effectively prevented CFA-induced orofacial mechanical allodynia at all five time points, including day 1 (acute effect), as evidenced by increased mechanical thresholds compared to the inflamed group without LLLT and the 2.5 J/cm^2^ group. Interestingly, the 5 J/cm^2^ dose demonstrated greater efficacy following a U-shaped curve. All doses reduced plasma extravasation, neutrophil infiltration and MPO activity, with 5 and 10 J/cm^2^ showing more pronounced effects compared to 2.5 J/cm^2^. Notably, leukocyte counts were significantly reduced at 5 and 10 J/cm^2^, while no significant reduction was observed with 2.5 J/cm^2^.

LLLT has gained interest as a novel treatment for TMJ arthritis, with studies showing its biomodulatory effects, promoting tissue regeneration and healing by regulating inflammation and immune responses [23, 24]. Our data corroborates these recent findings.

An intrinsic modulation of pain can also occur in the peripheral nervous system, mediated by an interaction between immune cells (inflammatory pattern) and the terminals of sensory neurons [25]. Studies have demonstrated that LLLT hinders the activity potential age and conduction of nociceptive flags in essential afferent neurons [26]. Along these lines, laser treatment represses the conduction of C filaments. It expands oxygenation and lymphatic waste, which helps with discomfort after the initial few minutes of tissue irradiation [26].

LLLT utilizes low-intensity light energy with wavelengths capable of penetrating biological tissues to modulate various physiological processes [27, 28]. It has been shown to influence the synthesis, release and metabolism of signaling molecules crucial to analgesia [29]. However, the precise mechanism of how LLLT acts at cellular levels must still be fully understood.

Cell photoreceptors consume laser light such as hemoglobin, myoglobin and cytochrome c oxidase, a protein inside the mitochondria that builds adenosine triphosphate (ATP) creation and diminishes oxidative pressure [27]. Further, LLLT increases beta-endorphins, lymphatic flow, blood supply and tissue oxygenation; shifts metabolism from anaerobic to aerobic pathways; and induce muscle relaxation. In contrast, it reduces the release of histamine, swelling and pain-related substances, bradykinin, and the production of acid metabolites, which stimulate the pain receptors and increase the duration of inflammation [29, 30].

Studies suggest that PBM reduces pro-inflammatory cytokines like TNF-α and IL-6 while promoting anti-inflammatory mediators such as IL-10. This modulation contributes to decreased pain sensitivity and improved tissue recovery, supporting its use as a therapeutic approach for managing pain and inflammation [26].

In our protocol a 2.5 J/cm^2^ laser dose at the late phase of CFA-induced inflammation reduced plasma extravasation in TMJs. In contrast 5 and 10 J/cm^2^ laser doses reduced Evans blue dye after the first laser application. A previous study verified a similar effect of LLLT on plasma extravasation during TMJ inflammation induced by formalin injection [31]. This reduction in plasma extravasation may have been due to the inhibition of cyclooxygenase, an isoenzyme responsible for the production of prostaglandins [32].

MPO activity in the TMJ synovial fluid was also reduced by LLLT. A more significant reduction in MPO was observed at 1 and 3 days with all laser doses used. In addition, at LLLT at 5 and 10 J/cm^2^, there were no differences in the MPO levels of the TMJ fluid of rats submitted or not to inflammation. Myeloperoxidase is a hemoprotein produced by neutrophils and monocytes that participates in various physiological and deleterious processes [33]. It is responsible for generating hypochlorous acid and certain drugs and toxins through the oxidation of endogenous compounds. This enzyme catalyzes the arrangement of responsive oxidants to battle attacking pathogens and assumes a focal role in the innate immune system [34]. Still, these reactive oxidants have been associated with deleterious effects that cause oxidative injury in inflammatory diseases [35].

The primary product of MPO action, hypochlorous acid, generates secondary products such as reactive oxygen species (ROS) with broad biological actions in events such as apoptosis and inflammatory processes. There is evidence confirming the participation of LLLT in reducing ROS production during tissue injury [18]. Some studies have proposed this effect as a mechanism of reducing the transcription of nuclear factor kappa B and the interleukins IL-1B and IL-6 [36], which accelerate capillary hydrostatic pressure, edema resorption and the elimination of inflammatory mediators.

The positive effects of LLLT in the acute inflammatory phase are related to the stimulation of respiratory chains and generate more effective ATP production. This effect also reduces ROS production at the site of the trauma [37]. The present results indicate LLLT’s effects in reducing MPO, mainly in acute phases of TMJ inflammation, which is probably associated with a decrease in ROS release, suggesting an antioxidant activity of LLLT and a modulation of the redox state, accelerating the recovery of injured tissue. Importantly, this study used low laser doses which induced a reduction in inflammation. Corroborating this idea, Silveira* et al*. [38] (2016) evidenced the prevention of oxidant markers in an animal model of induced acute muscle trauma using laser treatment with doses of 3 and 5 J/cm^2^ initiated 2, 12 and 24 h after the trauma.

In the first phase of an inflammatory process, neutrophils are the first leukocytes to be recruited to the site and can eliminate pathogens through different mechanisms. Articular crippling is specifically connected with the relocation of neutrophils into the joint cavity [39]. The present CFA-induced joint inflammation evidenced an increase of neutrophils in TMJ fluid at 1, 3, 7 and 10 days of inflammation, with higher values during the first and third days after the beginning of the inflammatory response. LLLT decreased this inflammatory infiltrate after 10 days of LLLT 2.5 and 5 J/cm^2^ doses and reversed neutrophil cells to normal levels with a laser dose at 10 J/cm^2^ in all the periods analyzed. Since MPO is stored in neutrophils, the reduction in MPO activity in this study can be due to the decreased influx of neutrophils. Based on the conditions of this study, a single dose of 10 J/cm^2^ immediately after induced temporomandibular inflammation proved to attract neutrophils effectively, demonstrating that the light conveyance technique toward the start of the inflammatory course is urgent to set off an upgraded cell reaction. LLLT 2.5 and 5 J/cm^2^ positively affected TMJ tissue but could not extinguish the inflammatory process. de Loura Santana *et al*. [40] (2016) demonstrated that LLLT can adjust the creation of inflammatory infiltrate in diabetic injuries, prompting a progressively adjusted reaction transiting from fast neutrophil invasion to a diminished influx, with a single application of 4 J/cm^2^ in the immediate postoperative period. In this study, neutrophil levels returned to normal after 1 and 3 days of laser treatment.

Regarding total leukocytes in the TMJ during the inflammatory process, these cells increased after CFA-induced inflammation in rats without LLLT and with LLLT at 2.5 and 5 J/cm^2^. The 2.5 J/cm^2^ dose could not reduce this inflammatory infiltrate in tissue, and the 5 J/cm^2^ dose decreased after 10 days. However, the 10 J/cm^2^ dose extinguished the inflammatory effects during all experimental times. Since the relocation of leukocytes from the blood dissemination to the harmed tissue is the essential stage in the inflammatory process and neutrophils assume a fundamental role in hyperalgesia, treatments that restrain the movement of neutrophils to the inflammatory event might be an option for control of pain. Thinking about this reality, this examination demonstrates that LLLT is a powerful alternative for controlling orofacial pain of TMD with articular components.

In this context, strategies that inhibit the excitation and/or sensitization of primary afferent nociceptive neurons, as well as prevent central events, could be beneficial for managing temporomandibular disorders. In the present study, we investigated the effects of LLLT irradiation on the TMJ of rats, focusing on both the initial inflammatory pain response and a persistent condition in a CFA-induced inflammation model, a well-established model of acute and chronic pain [41]. This point highlights the importance of this study by addressing the effect of LLLT in a dose-response curve and in acute, chronic, and transitional pain conditions, which are characteristic features of temporomandibular disorders [42].

It is important to emphasize that the results obtained are related to pain of inflammatory origin. In this context, acute conditions affecting the TMJ would be good candidates for LLLT treatment at the doses studied, such as arthralgias secondary to trauma, degenerative and/or rheumatic inflammatory diseases and displacements during the acute phase with the presence of pain. Chronic pain involving processes like central sensitization, neuronal convergence and/or dysfunction in pain-modulating pathways, which are not associated with peripheral inflammation, may not yield the same results, as LLLT effectively reduces peripheral sensitization.

This study has limitations that should be considered. Using an animal model poses challenges in translating findings to humans especially regarding dosage as clinical doses vary widely (1–112 J/cm^2^). Future studies are needed to determine the optimal dose for humans. Additionally, this study focused on short-term effects leaving long-term efficacy and safety unexplored particularly for chronic conditions. The young age of the animals may also limit the generalizability of the results. While a dose-dependent effect of PBMT was observed further research is essential to optimize dosing and account for individual variability and the complexity of TMD.

## 5. Conclusions

This study demonstrates that LLLT at doses of 5 and 10 J/cm^2^ is more effective than 2.5 J/cm^2^ in mitigating nociceptive responses in a rat model of persistent TMJ inflammation. The findings indicate that LLLT not only prevents mechanical allodynia following CFA-induced inflammation but also alleviates persistent nociception and modulates inflammatory biomarkers. Notably, the 5 J/cm^2^ dose produced the greatest increase in mechanical thresholds across all time points, suggesting that a relatively low laser dose can provide effective analgesia. These results underscore the dose-dependent nature of LLLT and its potential as a non-invasive analgesic approach. However, further research is necessary to refine optimal dosing parameters, evaluate long-term therapeutic effects and address translational challenges for clinical application in human populations.
